# Tuning of Water Vapor Permeability in 2D Nanocarbon-Based Polypropylene Composite Membranes

**DOI:** 10.3390/nano15010011

**Published:** 2024-12-25

**Authors:** Glykeria A. Visvini, Georgios N. Mathioudakis, Amaia Soto Beobide, George A. Voyiatzis

**Affiliations:** 1 Foundation for Research and Technology-Hellas (FORTH), Institute of Chemical Engineering Sciences (ICE-HT), Stadiou Str., GR-265 04 Rio-Patras, Greece; gvisvini@iceht.forth.gr (G.A.V.); mathioy@iceht.forth.gr (G.N.M.); asoto@iceht.forth.gr (A.S.B.); 2 Department of Physics, University of Patras, GR-265 00 Rio-Patras, Greece

**Keywords:** nanocomposites, 2D carbon-based nanomaterials, breathability, polypropylene, graphene

## Abstract

This work focuses on the incorporation of 2D carbon nanomaterials, such as graphene oxide (GO), reduced graphene oxide (rGO) and graphene nanoplatelets (GNPs), into polypropylene (PP) via melt mixing. The addition of these 2D carbon nanostructured networks offers a novel approach to enhancing/controlling the water vapor permeable capabilities of PP composite membranes, widely used in industrial applications, such as technical (building roof membranes) or medical (surgical gowns) textiles. The study investigates how the dispersion and concentration of these graphene nanomaterials within the PP matrix influence the microstructure and water vapor permeability (WVP) performance. The WVP measurements were conducted via the “wet” cup method. The presence of either GO, rGO or GNPs in the new polyolefin composite membranes revealed 6- to 7-fold enhanced WVP values compared to pristine PP. This improvement is attributed to the nanoindentations created at the interface of the carbon nanoinclusions with the polymer matrix in the form of nanopores that facilitate water vapor diffusion. In the particular case of GO and rGO, residual oxidative groups might contribute to the WVP as well. This is the first study to compare GO, rGO and even GNP inclusions under identical conditions, providing deeper insights into the mechanisms driving the observed improvements in WVP performance.

## 1. Introduction

Polymeric membranes are selective materials used in a wide range of applications that require separation processes, from water filtration and purification to industrial separation. An important property that polymeric membranes can exhibit is water vapor permeability (WVP), which makes them “breathable”. Breathability is defined as the ability of membranes to allow the diffusion of water vapors through them and at the same time prevent the penetration of water in liquid state [1,2]. This can be accomplished by various methods, including the incorporation of micro/nanofillers into the polymer matrix [1,2,3,4,5]. In this case, a micro/nanopore network can be formed, allowing water vapor to permeate through the membrane. The polymeric composite membrane can be stretched uniaxially or biaxially, either creating micro/nanopores and/or aligning or “cross-linking” them, respectively, in the composite, contributing to the increase in WVP [1].

Membranes that could provide selective blocking or permeability of water vapors find application in several sectors including dehydration of gaseous streams, moisture protection for sensitive electronics, breathable textiles and humidity control in buildings. In the latter case, new “breathable” composite polymeric membranes could be applied either (i) as building roof membranes, which allow water vapor to permeate through them and avoid the co-existence of moisture, and/or (ii) as vapor control layers (VCLs), which are capable of preventing water vapor generated on the building surface from freely diffusing towards the roof (wall, floor) and minimizing heat loss through convection.

Stretched membranes reinforced with CaCO_3_ particles are conventionally used to develop microporosity sufficient to impart breathability characteristics. On the other hand, 1D and 2D graphene-based nanomaterials, such as carbon nanotubes (CNTs) or graphene oxide (GO), are anticipated to improve WVP in polymer membranes. The ability of both CNTs and GO to allow rapid diffusion of water vapor through them is a promising implication for the development of breathable composites [4,5,6,7,8]. More specifically, the presence of the CNTs in PP composites, combined with the occurrence of the β- and α-PP crystalline phase, exhibited significantly enhanced WVP values [3]. The importance of crystalline-phase alterations within the polyolefinic matrix, with the β-phase providing higher permeability characteristics compared to the α-phase, was highlighted. The presence of CNTs further contributed to this enhancement by promoting the formation of nanochannels within the polymer structure, facilitating the diffusion of water vapor. These results also prompted the involvement of two-dimensional graphene nanomaterials, such as GO, rGO and GNPs, for eventual optimization of WVP properties in composite membranes. In parallel, graphene-based polymeric nanocomposites are proposed as membranes with high water permeability and near-impermeability to gases [9]. For instance, there are several studies on self-standing GO films where it is argued that the presence of different oxidizing functional groups (-OH, -C=O, -COOH, etc.) affects the distance between graphite sheets and, consequently, the water vapor permeability [9,10,11,12]. A recent review [13] underscores the potential of GO membranes in vapor permeation. Several models, including water diffusion (absorption–diffusion model) [14] or transport of condensed water (absorption–pore flow) [15] through its angstrom channels, have been evaluated to explain the observed selectivity. On the other hand, rGO, which results from the partial removal of the oxidizing groups in GO, is anticipated to exhibit barrier properties; however, in many cases, the remaining oxidizing functional groups and the many structural defects may result in notable water permeation [16]. Similarly, in GNPs, which could be considered ideal reinforcements for impermeable membranes due to their high aspect ratio and specific surface area [17], the WVP is significantly influenced by their specific structural characteristics such as the lateral size of their platelets [18].

The main scope of this study is to thoroughly examine and evaluate these three carbon-based 2D nanomaterials as reinforcements for water vapor breathable (or impermeable) polymeric membranes. In this context, polymeric composites consisting of i-PP polymeric matrix and graphene-based 2D nanomaterials, such as GO, rGO or GNPs, were developed and analyzed. The focus is on understanding how these nanomaterials influence the properties of the membranes, particularly in terms of water vapor permeability. iPP is a semi-crystalline polymer that, with the incorporation of nanoinclusions and appropriate processing, can lead to the improvement of the microporosity of the polymer matrix, thereby enhancing the WVP of its nanocomposites [19,20,21,22,23]. This property is expected to be further optimized with the incorporation of carbon-based 2D nanomaterials, which are anticipated to contribute to the overall performance of the membranes. This point-to-point comparison under similar composite preparation conditions could provide direct feedback in case breathability properties arise.

## 2. Materials and Methods

### 2.1. Materials

Graphene oxide (GO) was purchased from Abalonyx (Oslo, Norway, CAS: 18002, dry powder < 100 mesh). The reduced GO (rGO) was synthesized by the thermal reduction of the GO from Abalonyx. Specifically, a small amount of GO was heated in an oven at 50 °C overnight and kept there additionally for 2 h at 80 °C, for 1 h at 100 °C, for 1 additional hour at 150 °C and, finally, for 30 min at 200 °C. The GNPs were purchased from Thomas Swan (Consett, UK, CAS: SP 8099B, lateral size: 5 μm; thickness: 5–7 nm). The powdered form of polypropylene (ECOLEN^®^ HZ40S polypropylene homopolymer) was a product of the Hellenic Petroleum S.A. provided by Plastika Kritis S.A. (Crete, Greece).

### 2.2. Membrane Preparation

Nanocomposite membranes containing PP loaded with GO, rGO and GNPs (0.25 to 2.5 wt.%) were prepared by melt mixing with the appropriate amounts of powdered PP in a twin-screw extruder (Thermoscientific-minilab twin extruder HAAKE MINILAB II with conical screws) at 200 °C and a screw speed of 100 rpm for 3 min. The pellets of the composite compounds were pressed into membranes via a hydraulic press at ~200 °C and 80 bars. Polymer membranes had a thickness of about 100 μm (Figure S1). These membranes were further annealed at 130 °C (this temperature was selected due to its ability to promote the β-crystalline phase in PP, which is associated with WVP enhancement under suitable conditions) for 30 min [3]. Table 1 lists the membranes prepared in this study, indicating loading of the 2D carbon-based nanomaterial utilized.

## 3. Experimental Techniques

### Characterization Technique

In order to measure water vapor transmission rate (WVTR), the wet cup method described by ASTM E96/E96M-10 [24] was used, based on a homemade device, which has already been described [24]. Briefly, a dish filled with distilled water is covered by the tested membrane and placed in a chamber in which a cartridge heater and two N_2_ inlets (one for dry N_2_ and another for N_2_ passing through water) control the temperature and humidity level, respectively, while an axial fan ensures air circulation. During the experimental procedure, the weight change in the complete test assembly is measured every 5 min by a computer-interfaced scale inside the chamber. The experimental conditions for all examined membranes were 27 °C and 21% relative humidity (RH). Water vapor transmission rate (WVTR) is defined as the steady water vapor flow in a unit of time through a unit of area of a body, normal to specific parallel surfaces, under the specific conditions of temperature and humidity at each surface. A least-squares regression analysis of the change in mass as a function of time is used to determine the rate of water vapor transmission at a steady state. The WVTR was calculated at the steady-state region from the slope of the water mass loss as a function of time normalized to the area of the tested membrane [4,25,26]:

(1) $$\mathrm{WVTR}=\frac{\mathrm{water}\;\mathrm{mass}\;\mathrm{loss}}{\mathrm{time}\;\times \;\mathrm{area}\;}=\frac{\mathrm{flux}}{\mathrm{area}}$$

with units of g·m^−2^·min^−1^. Since the thicknesses of the membranes varied, the WVTR was normalized to membrane thickness, l, in order to obtain the specific water vapor transmission rate (l × WVTR) with units of μm·g·m^−2^·min^−1^.

The ATR-FTIR (attenuated total reflectance–Fourier transform infrared) spectra of solid samples were recorded on an Alpha-II Diamond ATR Spectrometer from Bruker Optics GmbH.

Scanning electron microscopy (SEM) (Zeis SUPRA 35 VP-FEG instrument operation at 5–20 kV) was used to examine the morphology of pristine GO, rGO and GNPs. SEM images of cryogenic cross-sections of the composites membranes were also examined.

AFM images were acquired by a peak force tapping mode with a Dimension Icon AFM (Bruker Corporation, Billerica, MA, USA). ScanAsyst-Air probes (stiffness 0.2–0.8 N/m, frequency ~80 kHz) were employed for morphology and thickness evaluation. Thermogravimetric analysis (TGA) measurements of the pristine GO, rGO and GNPs were performed using a TA 55 model, at a heating rate of 10 °C/min under nitrogen N_2_ conditions and a flow rate of 25 mL/min.

X-ray diffraction (XRD) was used to identify the crystalline phase of all composites. XRD measurements were carried out by using a Bruker D8 Advance diffractometer equipped with a Cu lamp (λCuKa = 1.54046 Å) at a scanning rate of 0.5°/min over a range of 5–30° (2θ).

Differential scanning calorimetry (DSC) measurements were carried out on a Q100 unit (TA Instruments) equipped with a liquid N_2_ cooling system. For the study of melting and crystallization behavior of net polymer and nanocomposites containing GO, rGO and GNPs, the samples were heated up to 200 °C with a heating rate of 10 °C min^−^^1^. All measurements were taken in a nitrogen atmosphere (50 mL/min). Data were obtained from the first heating cycle that referred to the actual state of the prepared composites.

X-ray photoelectron spectroscopy (XPS) analysis was performed in an ultra-high-vacuum (UHV) system equipped with a SPECS Phoibos 100-1D-DLD hemispherical electron analyzer and a non-monochromatized dual-anode Mg/Al X-ray source. The photoelectron spectra were recorded using the MgKα with a 1253.6 eV photon energy non-monochromatized source (300 W) and an analyzer pass energy of 10 eV, giving a full width at half maximum (FWHM) of 0.85 eV for the Ag3d5/2 line. The acquisition and fitting were realized with the commercial software SpecsLab Prodigy (Specs GmbH, Berlin, Germany).

## 4. Results and Discussion

### 4.1. Characterization of 2D Carbon-Based Nanomaterials

The morphological, structural, crystallographic and thermal properties of the 2D carbon-based nanomaterials (GO, rGO and GNPs), used to prepare the PP nanocomposite membranes, were investigated through scanning electron microscopy, AFM, ATR-FTIR spectroscopy, X-ray diffraction and Thermogravimetric analysis (TGA), respectively.

**Figure 1:**
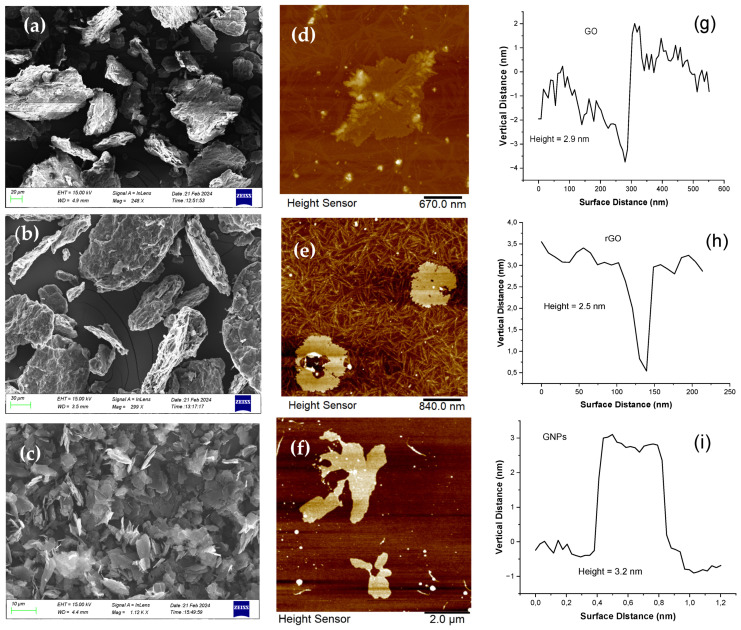
SEM (left column) and AFM (middle column) images of GO (**a**,**d**), rGO (**b**,**e**) and GNPs (**c**,**f**), respectively. The corresponding AFM height profiles are shown in (**g**–**i**).

The morphology of GO, rGO and GNP samples was characterized using SEM and AFM analysis (Figure 1). Based on SEM images, the lateral dimensions of GO were found to be between 15 and 50 μm, while rGO presents an approximately similar morphology and dimensions; the SEM image did not reveal any thinner sheet morphology of rGO compared to GO nor did it show a reduced distance between its layers. GNPs took the form of flakes (multilayer graphene platelets) with a typical lateral size of 5 μm. Figure 1 also shows the typical AFM height images of GO, rGO and GNPs; the GO sheets have a height of ≈3 nm, which is typical for GO flakes, while the average thickness of the rGO and GNP nanosheets was about 2.5 nm and 3.2 nm, respectively.

**Figure 2:**
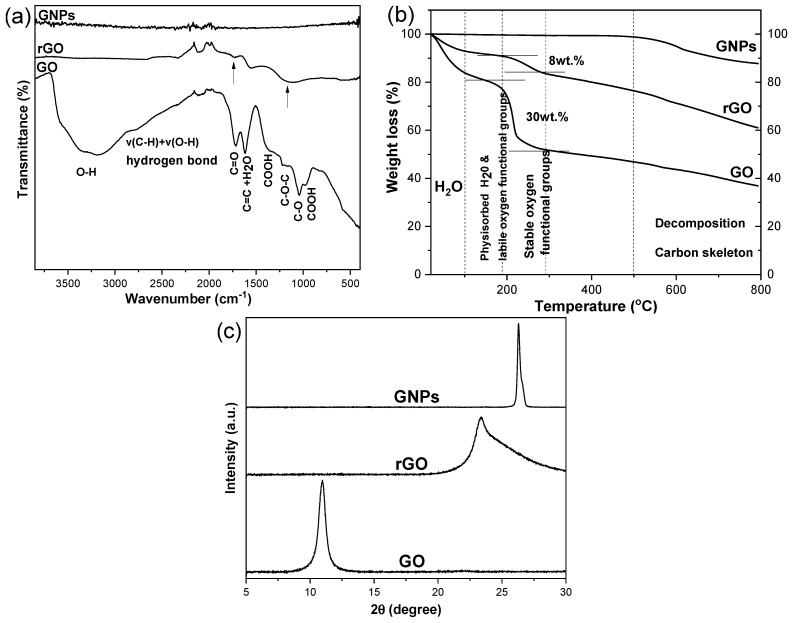
(**a**) ATR-FTIR spectra, (**b**) XRD patterns and (**c**) TGA thermographs of pristine GO, rGO and GNPs.

ATR-FTIR spectra (Figure 2a) and XRD patterns (Figure 2b) contributed to the identification of the chemical composition. In the ATR-FTIR spectrum of GO, characteristic peaks were noticed that related to its oxidizing functional groups (-OH, -COOH, C=O, -C-O and C-O-C). The assignment of these peaks is provided in Table 2. The peaks of the -COOH groups were detected at 970 and 1380 cm^−1^, whilst the peaks at 1040 cm^−1^ and 1220 cm^−1^ were assigned to the -C-O and -C-O-C groups, respectively. The peak at 1720 cm^−1^ corresponded to the carbonyl groups (C=O). It is evident that after the thermal reduction of GO to rGO, most of the peaks corresponding to the oxidizing functional groups decreased in intensity; however, partial and not complete reduction was noticed, as indicated by the persistence of the peak at ~1720 cm^−1^ (C=O) and in the 950–1300 cm^−1^ region (Figure 2a). In addition, as was anticipated, no absorption peaks were observed in the ATR-FTIR spectrum of GNPs.

The latter was further confirmed by XPS spectra obtained for the GNPs in which the presence of oxygen was not detected, as shown in Figure S2 (deconvoluted C1s spectra and the O1 core level spectra). From the peak areas of O1s and C1s divided by the relative sensitivity factors and the energy analyzer transmission characteristics, the % atomic concentration is 98.6 ± 0.1 carbon and 1.4 ± 0.1 oxygen for GNPs. On the other hand, the % atomic concentration for the GO is 69.9 ± 0.5 carbon and 30.1 ± 0.5 oxygen and for the rGO is 94.1 ± 0.5 carbon and 5.9 ± 0.5 oxygen. GO shows high percentages of oxygen, revealing the presence of several oxidizing groups with the ability to adsorb water. rGO shows some residual oxidizing groups after the reduction process achieved with considerably lower percentages of oxygen.

Figure 2b presents the TGA thermograms of all graphene/graphitic nanoinclusions. It is obvious that both GO and rGO are not thermally stable compounds and can easily decompose or lose weight, even at temperatures below 100 *°C*. Weight loss in this temperature window is primarily due to residual water molecules. Up to 190 °C, the slight weight loss is due to the removal of a few physically adsorbed water molecules and unstable oxygen-containing functional groups. As the temperature increases to 250 °C, the relatively stable oxygen-containing functional groups begins to decompose. Subsequently, the significant weight loss observed at temperatures between 100 °C and 250 °C is due to the removal of functional oxidizing groups in GO. Thus, from the TGA thermogram, we can derive the information that GO contains about 30 wt.% oxidizing groups, while in rGO, which has been obtained from the thermal reduction of the same GO, a percentage of ~8 wt.% oxidizing groups remains. Therefore, during its thermal reduction to rGO, GO loses a significant percentage of its oxidizing functional groups. In the case of GNPs, it is evident that they are thermally stable up to 500 °C. From 500 °C onwards, a rapid mass loss begins, which can be mainly attributed to the decomposition of the carbon skeleton of the GNPs [27,28]; this is also discernable for GO and rGO. A note is made of the fact that a bulk technique, TGA, and a surface technique, XPS, give such comparable results.

**Table 2:** Functional groups and ATR-FTIR peak position present in GO [29,30,31,32,33,34].

Wavenumber (cm^−1^)	Functional Groups Assignment
3000–3500 (broad)	O-H
2773	v(C-H)+v(O-H)hydrogen bond
1720	C=O stretching
1600	H_2_O (1616 cm^−1^)
	C=C (1580 cm^−1^) graphene layers
1380	COOH
1220	C-O-C
1040	C-O
970	COOH

Finally, the XRD patterns corresponding to the three components (GO, rGO and GNPs) are depicted in Figure 2c, showing reasonable differences considering their distinct structure and/or composition. XRD is one of the most comprehensive tools used to identify the type and quality of 2D carbon-based materials; using XRD, it is relatively easy to determine the composition and crystal structure of the material from the positions of the diffraction peaks. In the XRD pattern of GO, a characteristic peak appears at ~2θ = 11°, corresponding to the crystallographic plane (001) [32,33], and at an interlayer distance between the crystallographic planes of the crystal d~10.8 Å, which is due to the presence of various oxidizing functional groups attached to the carbon atoms of GO. The distance between the crystallographic planes, known as d-spacing, is calculated based on Bragg’s equation. The XRD pattern of GNPs presents a sharp peak, indicating a high degree of crystallinity at a higher diffraction angle, ~2θ = 26.3°. This peak corresponds to the (002) crystallographic plane. This peak is similar to that of graphite, confirming its stacked structure with a distance between the crystallographic planes of the crystal of d~3.4 Å. Essentially, comparing the XRD patterns of GO and GNPs, it is evident that the presence of various oxidizing groups in GO, such as hydroxyls (C-OH), carbonyls (C=O) and epoxides (C-O) on the basal plane and carboxyl acids (O-C=O) on the edges of GO, results in an increased interlayer distance between the graphene sheets, from 3.4 Å in graphite to 8 Å in GO. In the XRD pattern of rGO, a broad peak centered at ~23.4° appears, which corresponds to the (002) crystallographic plane, indicating a poor ordering of the sheets along the stacking direction. The distance between the crystallographic planes is slightly larger in rGO (3.8 Å) than in graphite (3.4 Å), suggesting the presence of some residual surface oxidizing groups in rGO. When GO is heated, to begin with, gradual dehydration takes place, while extended heating results in the reduction of GO. The partial reduction of GO, which has been revealed though ATR-FTIR, is also confirmed through XRD. In particular, the main diffraction peak of GO at ~2θ = 11° disappears in the rGO pattern with the simultaneous generation of the broad peak at ~23.4° [26,34,35].

Overall, we observe distinct features among the three different 2D carbon-based nanomaterials. All of the above results and conclusions from the characterization techniques of the graphene structures are summarized in Table 3.

### 4.2. Characterization of 2D Carbon-Based Nanocomposite Membranes

In a previous study related to PP/CNT composites, we suggested that the crystallization behavior of the membranes and the crystallographic phase is among the most important features to consider, regardless of their major or minor effect on WVP [3]. In this context, both DSC and XRD techniques were used collaboratively to characterize the crystalline structures present in the polymeric nanocomposites, comparing the crystalline behavior in each composite and evaluating if the 2D carbon-based nanofiller affects the crystalline properties of the polymeric matrix.

**Figure 3:**
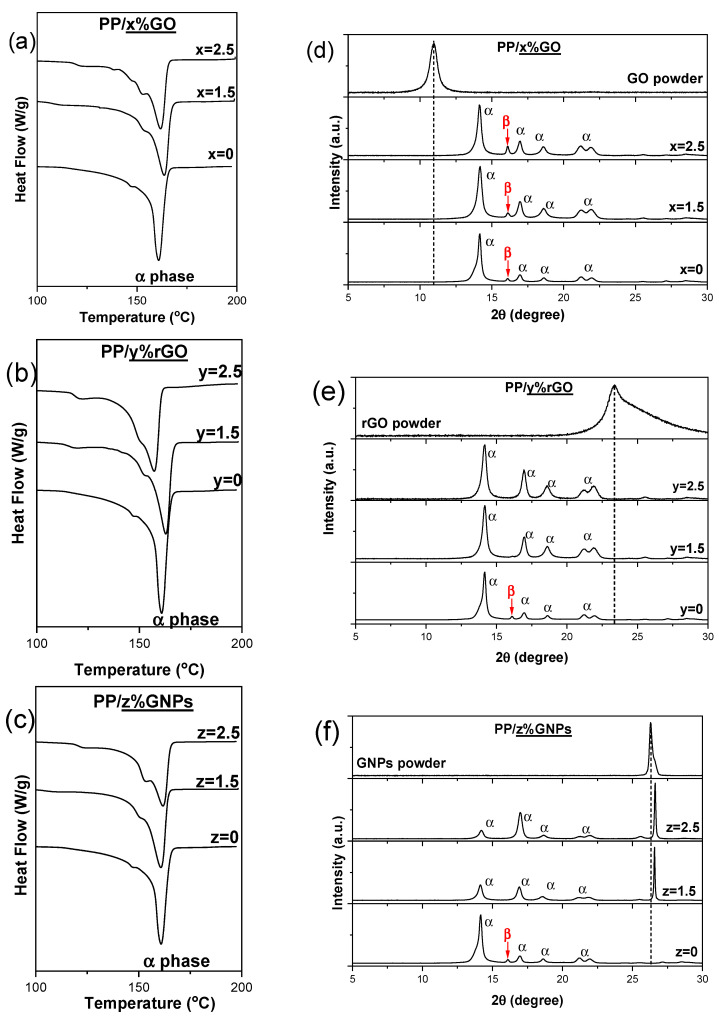
DSC thermograms (**a**–**c**) and XRD patterns (**d**–**f**) of PP/GO, PP/rGO and PP/GNP (0, 1.5 and 2.5 wt.%) nanocomposite membranes. Dotted lines indicate the position for the characteristic diffraction peaks corresponding to GO, rGO and GNPs respectively.

DSC thermographs of composite membranes PP/GO, PP/rGO and PP/GNPs (1.5 and 2.5 wt.%) are shown in Figure 3a–c. The procedure involved the heating of the membranes from −20 °C to 200 °C at a constant heating rate of 10 °C min^−1^. The typical endothermic melting peak of the α-crystalline phase of PP (melting point Tm~160 °C) is noticed in the DSC thermographs of pure PP polymer matrix, which become quite a lot broader with 2D carbon-based loading, indicating a wider crystallite size distribution in the nanocomposite membranes. For all of the nanocomposite membranes, a shoulder at around 155 °C appears prior to the main melting peak, most likely indicating the existence of different perfection degrees of α-crystals [36].

A similar characterization of the crystallization phases was also attempted through XRD analysis (Figure 3d–f). All samples showed diffraction peaks at 2θ = 14.2°, 17°, 18.6°, 21.2° and 22°, which correspond to the respective crystalline lattice planes, α(110), α(040), α(130), α(111) and α(131) + (041), of the α-crystalline phase of the PP [36,37]. The XRD patterns corresponding to the different composites are quite similar. It is noticeable that in the case of net PP and mainly in all PP/GO compositions, a very small diffraction peak at 16^o^ attributed to β-phase crystallites in PP is observed. This indicates that the development of a small portion of β-phase is favored. Moreover, the relative intensity peaks corresponding to the α-phase differ in the composite containing GNPs compared to the other two composites. In semi-crystalline polymers like polypropylene, the α-phase can develop a preferred orientation during processing, where the polymer chains align along a particular direction. This alignment leads to anisotropy in the crystalline structure, causing certain crystallographic planes to become more oriented in specific directions. Consequently, the relative intensities of the diffraction peaks from various crystal planes can vary. The morphology and size of GNPs, which defer from those of GO and rGO, may be responsible for the variations in the relative intensity of the peaks associated with the α-phase in PP.

It is remarkable that the characteristic diffraction peaks corresponding to GO and rGO nanoinclusion, typically observed at ~11° and 23.4°, respectively, are absent in the XRD patterns of their corresponding nanocomposite membranes. Even at the highest loading (2.5 wt.% GO or rGO), these characteristic peaks do not appear in the XRD diagrams of the composites. In contrast, a significant difference is observed in the XRD diagrams of PP/GNP composites, where the characteristic diffraction peak of GNPs is clearly noticeable. At the same time, a slight shift in this peak towards higher diffraction angles, from 26.2° on GNPs to 26.6° in the PP/GNP composites, indicates that the graphene nanosheets undergo a degree of compression upon their inclusion through melt extrusion into the polymer matrix. In the case of GO and rGO, a certain degree of layer exfoliation could be considered, as their characteristic peaks disappear (or shift to lower diffraction angles) upon incorporation into the polymer matrix.

Additionally, the water wettability of the composite materials was estimated by the water contact angle (θ) measurement. The prepared PP/2D nanocarbon-based membranes presented values lower than 90°, as shown in Figure S3. The membrane PP/1.5 wt.% GNPs exhibited the most hydrophobic behavior compared to the other membranes and had the largest value of contact angle. The oxygen-containing functional groups in GO and the remaining ones in rGO give a more hydrophilic behavior to the corresponding membranes.

### 4.3. Underlying Interactions Inside the Composite

For a comprehensive understanding of the WVP properties of the composite membrane containing the three different fillers (GO, rGO and GNPs), it is essential to emphasize the key findings revealed through the above characterization.

**Figure 4:**
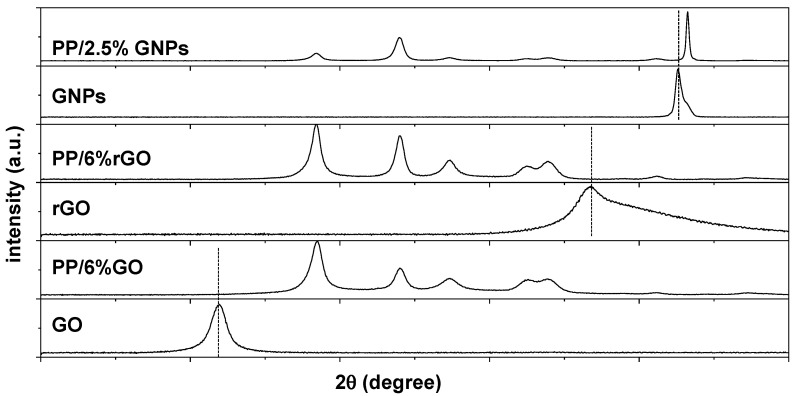
XRD patterns corresponding to PP/6 wt.% GO, PP/6 wt.% rGO and PP/2.5 wt.% GNP nanocomposite membranes. The XRD patterns of net GO, rGO and GNPs are depicted for comparison. Dotted lines indicate the position for the characteristic diffraction peaks corresponding to GO, rGO and GNPs respectively.

In the PP/GO and PP/rGO nanocomposite membranes, it was observed that the characteristic diffraction peaks of GO and rGO do not appear in the relevant XRD diagrams. Even at their highest loading (2.5 wt.%), the distinctive peaks that constitute the imprint of GO or rGO within the polymeric matrix are absent. In an attempt to reveal if the absence of the GO or rGO peak in the composite XRD pattern is due to the low GO or rGO fraction of carbon, new composites with higher loading of GO and rGO (i.e., PP/6 wt.% GO and PP/6 wt.% rGO) were also prepared, and their XRD patterns are presented in Figure 4. Nevertheless, despite the higher GO or rGO loadings, the XRD fingerprint of GO or rGO remains indiscernible.

In a further effort, the PP/6 wt.% GO membrane was also prepared following a film-casting approach (see Section S4). Film casting is a technique through which extensive heat treatment and shear stresses, which are developed during the melt-mixing extrusion technique adopted for the composites’ preparations, can be avoided. When the film-casting method was applied, the characteristic diffraction peak corresponding to GO nanoinclusion was just observable in the XRD pattern of the nanocomposite film (Figure S4). The slight differentiation between the two preparation methods may be attributed to the noninvasive nature of the film-casting process, which avoids the heat treatment and shear stresses involved in melt-mixing extrusion preparation. As a result, in the case of the film-casted composite, the GO filler is partly incorporated into the matrix in its original form, while its partial exfoliation continues to apply here to a certain extent.

**Figure 5:**
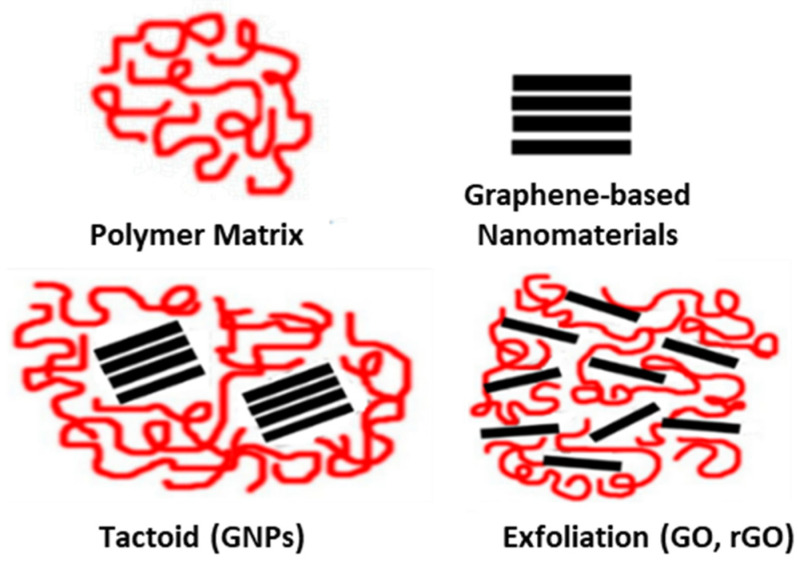
Schematic representation of the different types of composites formed based on the nature of interactions of the polymer with 2D carbon-based nanomaterials.

Furthermore, with the aim of clarifying the absence of the characteristic XRD peak of GO in the composites, small-angle X-ray diffraction (SAXS) measurements were also conducted for the PP/2.5 wt.% GO nanocomposite membrane. The purpose of the SAXS analysis was to reveal a potential exfoliation of the GO in order to understand the absence of its pattern in the XRD diffractogram of the nanocomposite membrane. SAXS measurements can be made at very small angles, typically in the range of 0.1 degrees to 5 degrees, while XRD starts at 5 or higher. This is the purpose of the SAXS measurements; due to exfoliation, the GO pattern should be shifted to lower angles that could be seen by SAXS. Once again, it was observed that the characteristic diffraction peak corresponding to the GO nanoinclusion does not appear in the SAXS pattern (Figure S5). Therefore, in the case of GO loading into the PP matrix, it is reasonable to assume that GO undergoes (partial) exfoliation. This could be attributed to the intense temperature stresses during molding and the shear stresses generated during mixing via the melt-mixing technique. In a conceptual design of this assumption, the GO is included in the PP/GO composite in the form of dispersed GO sheets within the polymer matrix. This also holds for the rGO case. On the contrary, in the PP/GNP composites, intact stacks of GNPs are included in the composite, forming a tactoid–polymer configuration (Figure 5).

### 4.4. Water Vapor Transport Through Hybrid Composite PP Membranes

**Figure 6:**
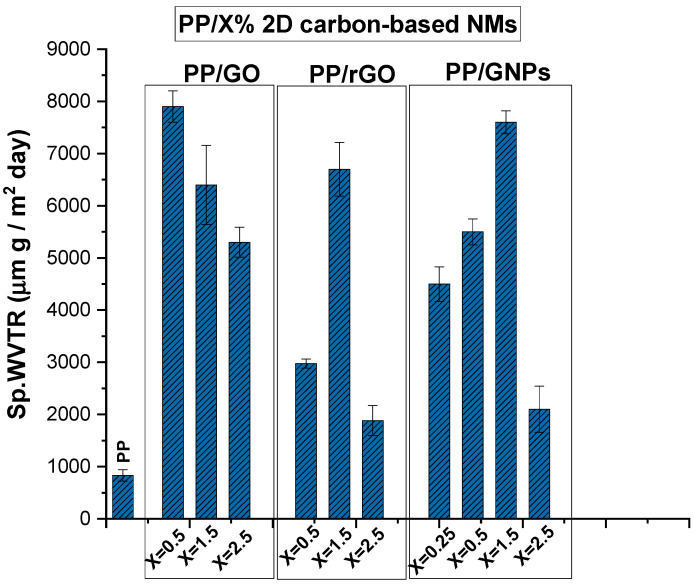
Sp.WVTR values for PP/x wt.% 2D carbon-based nanomaterials composite membranes.

The specific water vapor transmission rate (Sp.WVTR) values of PP nanocomposite membranes loaded with 2D carbon-based nanomaterials (GO, rGO and GNPs) have been evaluated and are depicted in Figure 6. The Sp.WVTR values have been measured at RT and 21% relative humidity (RH) following the wet cup method.

It was observed that all PP/x wt.% 2D carbon-based nanocomposite membranes (GO, rGO and GNPs) exhibit enhanced average values of Sp.WVTR compared to the values obtained for net PP polymer matrix membranes. Pristine PP membrane exhibits a significantly lower Sp.WVTR value due to its hydrophobic nature and low inherent porosity. In particular, for PP/GO nanocomposite membranes, containing 0.5 wt.% GO, the Sp.WVTR values increase considerably (~6–7 times) compared to the permeability obtained for pure PP. The increased WVP in the case of PP/GO nanocomposite membranes, even at low loadings, is probably due to the presence of the various oxidizing groups (-COOH, -OH, -C=O and -C-O-C) in GO (exfoliated or not) within the PP polymeric matrix. In particular, the oxidizing groups existing in the GO structure lead to defects and imperfections in the structure, creating a nonperiodic structure. This discontinuation in the structure, along with the uniquely large 2D surface of graphene, could be alleged as the factor that enables the rapid transport of water vapors through GO, thereby enhancing the permeability of polymeric nanocomposite membranes. All this is suported by having in mind that GO sheets consist of both oxidized and pristine regions. The pristine regions, forming the channel walls, are hydrophobic and atomically smooth surfaces, allowing water to traverse the interlayer spaces at high speed with negligible friction [9,38]. Simultaneously, the hydrophilic nature of GO, attributed to its oxidized groups, enables preferential water adsorption. The adsorbed water molecules are proposed to “hop” or “jump” between these groups, facilitating diffusion through the absorption–diffusion model [14] or transport of condensed water (absorption–pore flow model) [15] via angstrom-scale channels. Comparing the other 2D carbon-based nanomaterials with reduced (rGO) or absent (GNPs) oxidizing groups, we can say that PP/GNPs and PP/rGO present very similar Sp.WVTR values for loadings of 1.5 and 2.5% wt.%, as can be observed in Figure 6. The discrepancy arrives for 0.5 wt.% loading, with PP/rGO presenting somewhat lower Sp.WVTR values than the corresponding PP/GNPs. In this case, there is low loading of rGO and consequently very few remaining oxidizing groups that facilitate water adsorption and allow water molecules to “hop” from one group to another. The smooth, frictionless graphitic surface is the main driving force promoting water vapor transmission, together with the microporous formation at the interfaces with the polymeric matrix. The fact that PP/1.5 wt.% GNPs exhibit the best performance can be attributed to water passing upon the external surface at a high speed without any friction but also to the intrinsic dimensions of these specific GNPs (with typical lateral size of 5 μm); bigger dimensions most probably act as a barrier to water transmission rather than facilitate it. Of course, the disruption of the polymer’s microstructure, creating microporous formations at the interfaces of the 2D material with the polymeric matrix, thereby ensuring the water vapor permeability of nanocomposite membranes, is also considered. The maximum Sp.WVP for PP/rGO and PP/GNPs is observed at a nanoinclusion concentration of 1.5 wt.%. At higher loadings of all nanoinclusions, the permeability gradually decreases. This can be explained by the fact that, at a high concentration of nanoinclusions, their dispersion in the polymer matrix is hindered by the formation of aggregates, which obstruct the facilitation of water vapor permeability.

Furthermore, the effective transport channels for water permeation could be quantified [9]. Graphene sheets in graphene-based membranes are separated by an interlayer distance, d, determined by XRD. Taking into account that the electronic clouds around graphene sheets extend over a distance of a ≈3.5 Å, the above separation d translates into an ‘empty’ space of width δ = d − a, which is available for water to diffuse into (water vapor dimensions 1.4–2.4 Å). For GO, the “empty space” would be 7.3 Å, while 0.3 and 0 are calculated for rGO and GNPs, respectively. So, water could cross between the graphene layers only in the case of GO.

**Figure 7:**
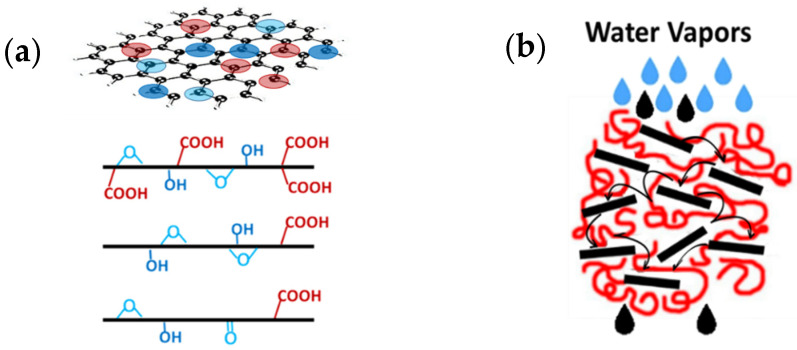
(**a**) Schematic representation of graphene and the various oxygen-containing groups; (**b**) sketch describing the hopping phenomenon of water vapor in the case of PP/GO and PP/rGO composites.

To summarize, in the above cases, the water vapor is initially bound through the oxidizing groups of GO and the few remaning groups in rGO (Figure 7a), and the water vapor “jumps” between these groups. At the same time, due to the hydrophobic nature of the graphitic structure of GO, the vapor passes through the interlayer channels at high speed. Thus, a hopping phenomenon of water vapor might occur (Figure 7b) with rapid transport through the polymeric composite. For rGO, which possesses less oxidized groups, instead, the rapid water transport through the interlayer spaces can be better described by the 2D nanocapillary model, similar to that proposed by Nair et al. [10].

**Figure 8:**
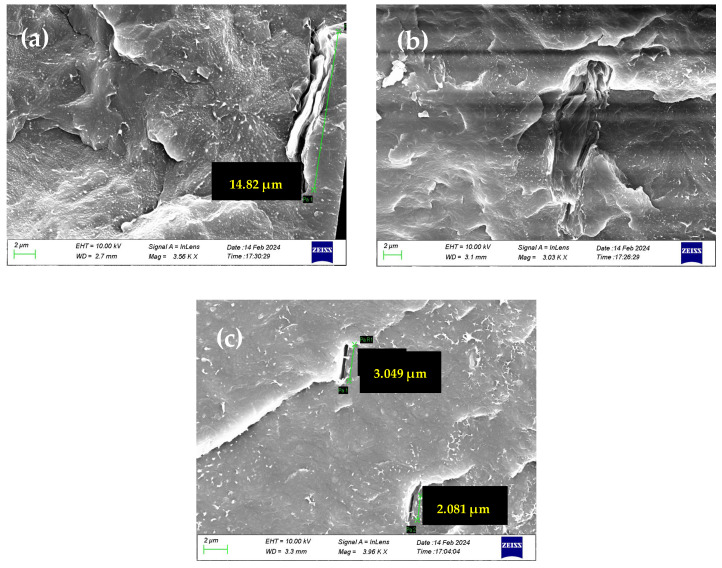
SEM images of (**a**) PP/1.5 wt.% GO, (**b**) PP/1.5 wt.% rGO and (**c**) PP/1.5 wt.% GNP nanocomposite membranes, and the pore generation occurring at the interface of the polymer matrix and 2D graphene nanostructures.

For GNPs, which lack oxidized groups, water traverses the slipping graphitic domains. The adsorption–diffusion model (adopted when oxidized groups are present) does not adequately explain the high permeability observed in PP/GNP membranes. Instead, the rapid water transport upon the external surface can better describe the Sp.WVTR values. In addition, the GNPs, which have not undergone exfoliation during melt mixing with the PP polymer matrix, retain a firm structure with a relatively small lateral size. As a result, the GNPs seem to disrupt the polymer’s microstructure, creating microporous formations at their interfaces with the polymeric matrix, thereby ensuring the water vapor permeability of PP/GNP nanocomposite membranes. This phenomenon probably cannot be excluded from the other composite membranes, regardless of the nature of the 2D carbon-based nanomaterial. This is confirmed by the SEM images depicted in Figure 8, which show the voids created at the polymer-inclusion interfaces, contributing to porosity development.

From the SEM images, we can discern the development of nanoporosity at the boundaries of all 2D carbon-based fillers, which can be related to the initially high Sp.WVTR (of the order of 8000 µm g/m^2^ day) compared to pure PP. The low affinity of polymer and 2D carbon-based inclusions can lead to the formation of “interfacial voids” and nanoindentations, which create pathways that facilitate permeability. This helps explain the increased water vapor permeability (WVP), regardless of the type of inclusion or the presence of oxidizing groups within the polymer matrix. It is evident that the distribution of nano- or microporosity generated by the inclusions plays a significant role in water vapor diffusion. In this context, a Kelvin effect that could decrease the partial pressure of the water at the above interfacial voids cannot be excluded. Moreover, it is important to note that the maxima in water vapor permeability can be constrained due to both the presence of aggregates at the high loadings of graphene 2D nanomaterials and the continuous consequence of the Kelvin effect, which can limit the maximum possible water vapor permeability at all loadings.

In Table 4, various values of Sp.WVTR achieved in the literature for polymeric membranes incorporating 2D nanomaterials or nanofillers are presented, along with those obtained in the present study. This table aims to directly compare the performance of the membranes developed in this study with others reported in the literature, though it is not possible to include all such examples.

**Table 4:** Summary of Sp.WVTR values for composites with 2D nanomaterial-based membranes or nanofiller-incorporated membranes in the literature.

Membrane	Conditions (T/RH)	Sp.WVTR (μm g/m^2^ Day)	Reference
GO-based freestanding	22 °C/85%	2315	[26]
PP	27 °C/21%	~800	This study
PP/0.5 wt.% GO	27 °C/21%	~7900	
PP/1.5 wt.% rGO	27 °C/21%	~6700	
PP/1.5 wt.% GNPs	27 °C/21%	~7600	
PP/MWCNTs	27 °C/21%	~1900	[3]
PP/β-nucleating agent/MWCNTs	27 °C/21%	~6000	
PP/40 wt.% TiO_2_	37.8 °C/36%	~4500	[39]
LLDPE/CaCO_3_ 55/45 ratio% (*w*/*w*)	38 °C/90%	~16,000	[40]
LLDPE/30 wt.% CaCO_3_/6 wt.% Al	37 °C/50%	6713.38	[41]
PVA/0.4 wt.% GO	23 °C/55%	12,000	[42]
Polyurethane/3 wt.% rGO	25 °C/80%	4000	[43]
PLA/nanocellulose fibers (NCFs) 90/10 (mass ratio)	23 °C/50%	5000	[44]
Epoxy/5 wt.% GNPs	38 °C/90%	~3200	[45]
Epoxy/5 wt.% boron nitride	38 °C/90%	~2000	

## 5. Conclusions

From the WVP measurements, it was observed that all PP/2D nanocarbon-based composite membranes exhibited enhanced Sp.WVTR values, approximately 6–7 times greater than those of the pure polymer matrix membranes. Specifically, maximum permeability was noticed at a concentration of about 0.5 to 1.5 wt.% (2D) nanoinclusion, while higher concentrations led to a significant decrease in permeability. Whereas it is plausible that some percentage of GO and rGO may undergo exfoliation, the enhanced water vapor permeability values obtained for the nanocomposites containing GO and rGO can be attributed both to the presence of/remaining oxidizing groups (such as carbonyls), as well as to a hopping effect of water vapor within the composite, which facilitates the penetration of water vapors. Additionally, the increased WVP values encountered for all of the 2D carbon-based nanocomposite membranes studied (PP/GO, PP/rGO and PP/GNPs) can be linked to the nano/microvoids that appear to be created at the interface with the polymer matrix. In the same context, a contribution to the rapid water vapor transport due to the frictionless, smooth and chemically inert graphitic structure of these 2D carbon-based nanomaterials at these interfaces cannot be ruled out.

## Figures and Tables

**Table 1:** List of samples prepared with PP as polymer matrix.

(2D) Carbon-Based Nanomaterial Loading				
GO (wt.%)	-	0.5	1.5	2.5
rGO (wt.%)	-	0.5	1.5	2.5
GNPs (wt.%)	0.25	0.5	1.5	2.5

**Table 3:** Summary results from characterization techniques applied.

Characterization Technique	GO	rGO	GNPs
SEM (lateral size, μm)	15–50	15–50	5–10
ATR-FTIR (oxidizing groups)	-OH, -COOH, C=O, -C-O, C-O-C	-C=O	-
XRD (d-spacing, Å)	10.8	3.8	3.4
TGA (oxidizing groups)	30%	8%	-
XPS (% atomic concentration)	C: 69.9 ± 0.5 O: 30.1 ± 0.5	C: 94.1 ± 0.5 O: 5.9 ± 0.5	C: 98.6 ± 0.1 O: 1.4 ± 0.1

## Data Availability

The raw data will be available from the corresponding author upon reasonable request.

## Supplementary Materials

The following supporting information can be downloaded at: https://www.mdpi.com/article/10.3390/nano15010011/s1, Figure S1. SEM image of cross-section of neat PP membrane. Figure S2. Deconvoluted C1s XPS spectra of (a) GO, (c) rGO and (e) GNPs, and O1s XPS spectra of (b) GO, (d) rGO and (f) GNPs. Figure S3. Contact angle images of (a) neat PP, (b) PP/1.5 wt.% GO, (c) PP/1.5 wt.% rGO and (d) PP/1.5 wt.% GNPs. Figure S4. XRD diagrams of (a) the PP/6 wt.% GO nanocomposite membrane prepared by the melt-blending technique in the twin-screw extruder, (b) the PP/6 wt.% GO nanocomposite membrane prepared by the membrane-casting technique, (c) the membrane of the pure PP polymer matrix and (d) the GO nanoinclusion. Figure S5. SAXS diagrams of the PP polymer matrix membrane, PP/2.5 wt.% GO nanocomposite membrane and GO nanoinclusion.
