# Supplementary material for: Tuning of Water Vapor Permeability in 2D Nanocarbon-Based Polypropylene Composite Membranes
Source: Nanomaterials (Basel). 2024 Dec 25;15(1):11. doi: 10.3390/nano15010011 (PMC11721603; doi:10.3390/nano15010011)
Supplement: Supplementary file 1 [file nanomaterials-15-00011-s001.zip › nanomaterials-3367846-supplementary.pdf]

nanomaterials

Supplementary Material

Tuning of Water Vapor Permeability in 2D Nanocarbon-Based Polypropylene Composite Membranes

Glykeria A. Visvini [1,2], Georgios N. Mathioudakis [1], Amaia Soto Beobide [1] and George A. Voyiatzis [1,*]

[1] Foundation for Research and Technology-Hellas (FORTH), Institute of Chemical Engineering Sciences (ICE-HT), Stadiou Str., GR-265 04 Rio-Patras, Greece; gvisvini@iceht.forth.gr (G.A.V.); mathioy@iceht.forth.gr (G.N.M.); asoto@iceht.forth.gr (A.S.B.)

[2] Department of Physics, University of Patras, GR-265 00 Rio-Patras, Greece

* Correspondence: gvog@iceht.forth.gr; Tel.: +30-2610-965253

1. SEM Images

Figure S1 shows a representative SEM image of the cross-section of neat PP and PP/1.5 wt.% GNPs membranes. The thickness of the membranes can be accurately calculated from the images. The thickness of all prepared membranes was approximately 100 μm.

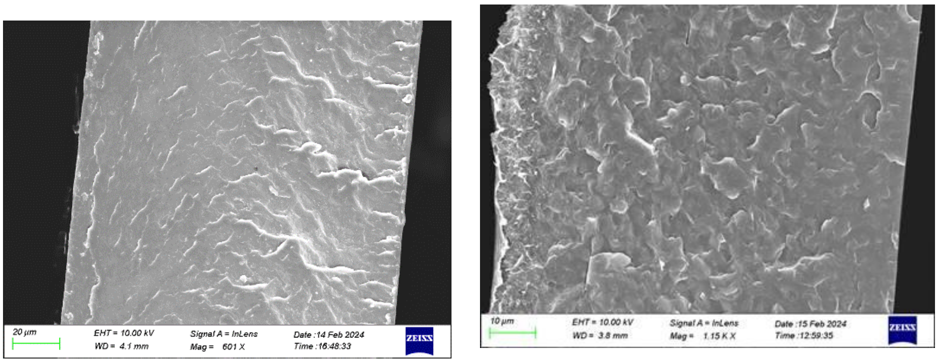

.

Figure S1. SEM image of the cross-section of neat PP (left) and PP/1.5 wt.% GNPs (right) membranes.

XPS Measurements: X-Ray photoelectron spectroscopy measurements were performed in order to investigate the chemical speciation and the relative atomic ratio of the 2D carbon based nanofillers GO, rGO and GNPs.

 https://doi.org/10.3390/nano15010011

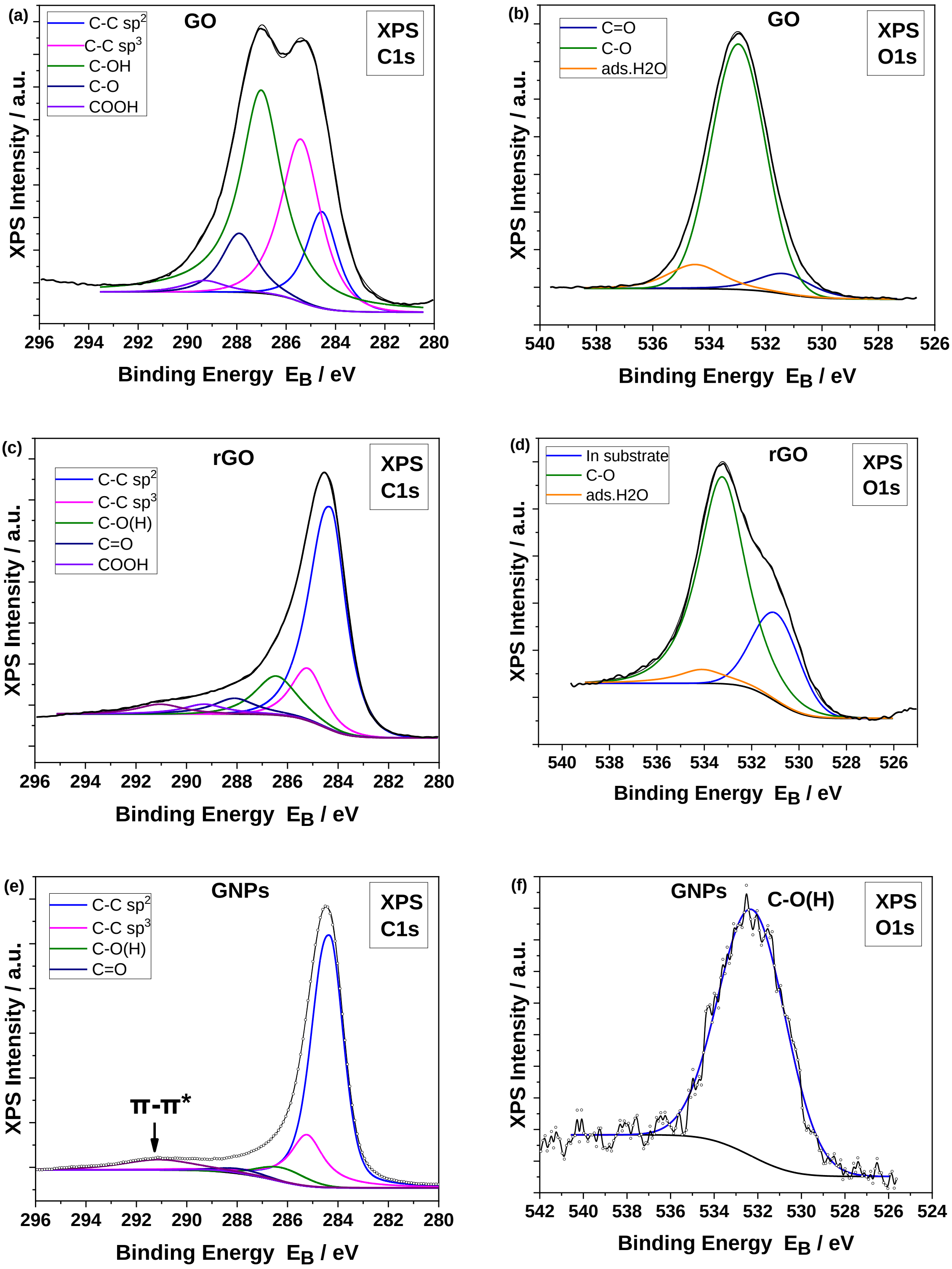

Figure S2 Deconvoluted C1s XPS spectra from **(a)** GO, **(c)** rGO and **(e)** GNPs and O1s XPS spectra from **(b)** GO, **(d)** rGO and **(f)** GNPs.

2. Contact Angle Measurements

Contact angle measurement provides information on the surface characteristics of membranes such as wettability, surface charge and tension, hydrophilicity, and interaction energy. The contact angle is the angle between the tangent line of the water droplet

on the surface and the baseline of the droplet. Figure S3 presents the contact angle measurements of net PP, PP/1.5 wt.% GO, PP/ 1.5 wt.% rGO and PP/ 1.5 wt.% GNPs membranes of. The membrane PP/1.5 wt.% GNPs exhibits the most hydrophobic behavior compared to the other membranes and has the largest value of contact angle. The oxygen-containing functional groups of GO and the remaining oxygen-containing functional groups of rGO give a hydrophilic behavior on the corresponding membranes.

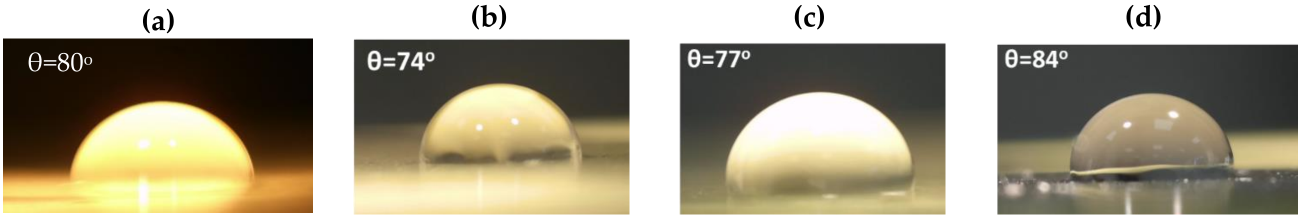

Figure S3. Contact angle images of **(a)** neat PP, **(b)** PP/1.5 wt.% GO, **(c)** PP/1.5 wt.% rGO and **(d)** PP/ 1,5 wt.% GNPs

3. PP/6 wt. %GO Film Casting

The PP/6 wt%. GO membrane was prepared by the film casting technique. The procedure followed for the preparation of the specific nanocomposite membrane was as follows: PP polymer and GO were solved in xylene under stirring at 105 °C until the suspension was homogenized. Afterwards, the solution was poured on a glass petri dish and evaporated by placing it in the oven 140 °C, w. In the XRD diagram of the specific nanocomposite membrane, it is obvious that the characteristic diffraction peak of the GO nanoinclusion can be distinguished (Figure S4b).

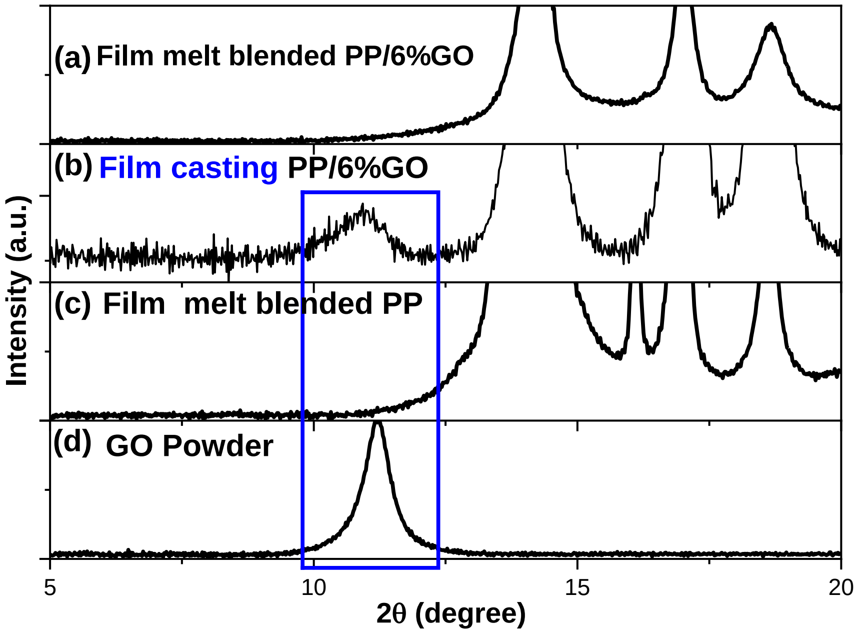

Figure S4. XRD diagrams of (a) the PP/6 wt.% GO nanocomposite membrane prepared by the melt blending technique in the twin screw extruder, (b) the PP/6 wt.% GO nanocomposite membrane prepared by the film casting technique, (c) the membrane of the pure PP polymer matrix and (d) the GO nanoinclusion.

4. Small Angle X-ray Diffraction Technique

The exfoliation of GO (or even the intercalation of pp into GO) was study for the PP/2.5 wt.% GO nanocomposite membrane via small angle X-ray diffraction technique SAXS (Small angle scattering) at IESL/FORTH (Institute of Electronic Structure and Laser, of the Foundation for Research and Technology). The SAXS analysis revealed that the characteristic diffraction peak associated with GO is absent in the SAXS pattern of the PP/2.5 wt.% GO nanocomposite membrane (Figure S5), suggesting a potential exfoliation trend.

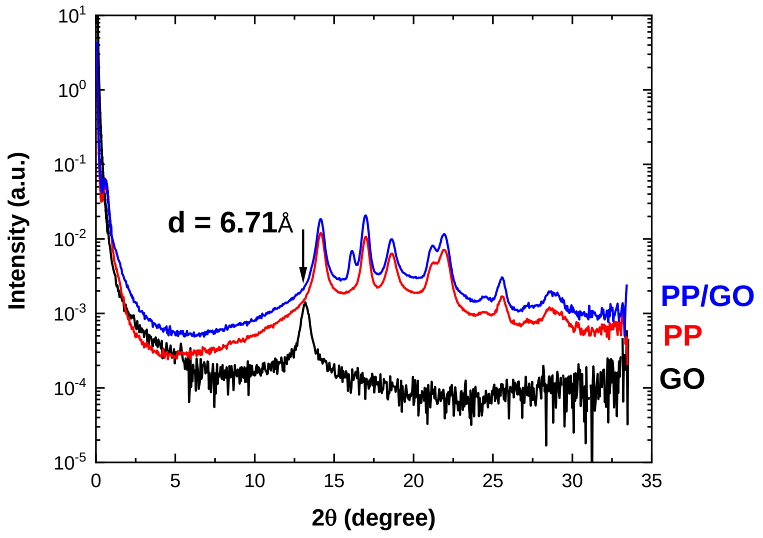

Figure S5. SAXS diagrams of PP polymer matrix membrane, PP/2.5 wt.% GO nanocomposite membrane and GO nanoinclusion.
